# A Dose of Smart Medicine to Make America Healthy Again: A $1 Trillion Win for Health, Innovation, and the Nation

**DOI:** 10.7759/cureus.83973

**Published:** 2025-05-12

**Authors:** Shaheen E Lakhan

**Affiliations:** 1 Medical Office, Click Therapeutics, Inc., New York, USA; 2 Department of Neurology, Western University of Health Sciences, Pomona, USA; 3 Department of Neurology, A.T. Still University School of Osteopathic Medicine in Arizona, Mesa, USA; 4 Department of Neurology, Morehouse School of Medicine, Atlanta, USA; 5 Department of Bioscience, Global Neuroscience Initiative Foundation, Miami, USA

**Keywords:** chronic disease, digital therapeutics, fda-regulated, healthcare cost savings, healthcare reform, maha initiative, prescription digital therapeutics, smart medicine, smartphone-delivered treatment, value-based care

## Abstract

Smart medicines are FDA-regulated, evidence-based, smartphone-delivered treatments used alone or alongside drugs. Unlike general health apps, these prescription digital therapeutics are authorized to make treatment claims and are already in use for conditions such as migraine (CT-132), insomnia (Somryst), and major depressive disorder (Rejoyn). They are poised to transform American healthcare. By delivering precision interventions directly into patients’ daily lives, they reduce the chronic disease burden, improve outcomes, and lower costs at scale. Drawing from my experience treating diverse populations across America, training future clinicians, shaping drug formularies, and developing digital therapeutics, I argue that smart medicine is the most promising lever for national health reform. If widely adopted in 2025, these technologies could save over $1 trillion annually while advancing healthcare access, restoring individual productivity, and reasserting U.S. leadership in medical innovation. Aligned with the goals of the Make America Healthier Again (MAHA) initiative, smart medicines support every major U.S. Department of Health and Human Services (HHS) priority, improving access, equity, and affordability of healthcare services for chronic diseases, behavioral health, and maternal care, and are thus uniquely positioned to close systemic healthcare gaps. This editorial proposes a federal initiative, Operation Smart Medicine, to accelerate the development, reimbursement, and nationwide deployment of smart medicines. Modeled on successful U.S. programs like Operation Warp Speed for COVID-19 and the HITECH Act for electronic health records, this initiative would enable smart medicines to support HHS/MAHA priorities and deliver scalable, value-based care. The technology exists, the evidence is emerging, and the time to align innovation with implementation is now.

## Editorial

From academic hospitals in Boston to rural clinics in Appalachia, I have witnessed firsthand the deep fractures in our healthcare system. Despite scientific breakthroughs and record-level spending, too many patients wait too long for care, face ineffective treatments, and navigate a system that is neither humane nor sustainable. As a physician, educator, policymaker, and developer of therapeutics, I have lived the contradictions, and seen the solution.

America does not need to work harder; it needs to work smarter. We must elevate what medicine means in the digital age and in the context of advancing AI technologies. Smart medicines, smartphone technologies that deliver FDA-cleared interventions through software, offer the AI-guided precision, scale, and cost-effectiveness to meet this moment [1]. Unlike general wellness apps or digital trackers, they deliver dose-based, regulated treatments that actively modulate physiology to achieve clinical outcomes. These treatments do not just improve disease; they engage behavior, modulate brain circuits, and restore function. They are rooted in validated clinical science and delivered through the platforms people already use: smartphones and intelligent systems that extend care beyond clinic walls.

This shift reflects a deeper truth: modern medicine must not only treat illness but also help individuals manage healthcare complexity, comorbidities, chronic conditions, cognitive load, and the behavioral inertia that keeps people stuck. Smart medicines do exactly that, meeting patients where they are with tools designed for daily life. By embedding health information and treatments into the smartphone devices patients already carry, we can also improve health education, close the gap between intent and action, and support treatment adherence.

In a post-pandemic world, with the U.S. Department of Health and Human Services (HHS) restructuring under the Department of Government Efficiency and the administration rallying behind the Making America Healthy Again (MAHA) initiative [2], the time has come to modernize healthcare through technologies that already exist and already work. The opportunity to act boldly is now.

In this editorial, I argue for the nationwide adoption of smart medicines, outline their financial and clinical advantages, and propose a federal initiative, Operation Smart Medicine, to ensure their rapid rollout from sea to shining sea.

A win for America’s health

Migraine is among the most common and disabling brain diseases worldwide, affecting nearly 40 million Americans and costing billions in direct and indirect expenditures. Many patients face limited access to neurologists and headache specialists due to a worsening provider shortage, particularly in rural and underserved regions. Meanwhile, existing drug treatments are often expensive, come with intolerable side effects, or lose effectiveness over time. CT-132 addresses these challenges, it is noninvasive, self-administered, side-effect-free, clinically validated, and recently granted FDA authorization [3]. For patients who have cycled through multiple ineffective or toxic medications, it offers an entirely new therapeutic pathway. For clinicians, it expands the treatment arsenal without increasing burden. For payers and policymakers, it delivers a scalable, value-based intervention in a field dominated by costly biologics and trial-and-error prescribing.

Smart medicines are certainly not limited to the management of migraine. They have been proven in randomized trials to treat major public health conditions such as type 2 diabetes [4], major depressive disorder [5], chronic insomnia [6], and chronic pain syndromes [7]. These digital therapies support recovery, not just symptom suppression, and are designed for use in daily life.

What differentiates smart medicines from other digital tools is their ability to actively modulate the human body’s physiology. These are not passive trackers or coaching apps. They deliver structured, regulated, dose-responsive interventions based on neurocognitive, emotional, behavioral, and physical mechanisms validated by robust scientific literature. For example, smart medicines can digitize, personalize, and scale highly effective face-to-face therapies for smartphone use, such as cognitive behavioral therapy for anxiety [8], musculoskeletal rehabilitation for joint injuries [9], or cognitive-linguistic rehabilitation for traumatic brain injuries and stroke [10]. These services, traditionally offered by psychologists, physical/occupational therapists, and speech-language pathologists, are often not widely accessible. Many digital therapeutics engage brain pathways associated with self-regulation, attentional control, reward processing, emotional regulation, and pain circuits [3,5,11], even targeting conditions historically considered “undruggable” by traditional pharmacotherapy. When paired with specific drugs, smart medicines can optimize outcomes, synergize effects, and potentially reduce the required dose while maximizing benefit and minimizing adverse effects. Smart medicine extends therapeutic effects into daily life with no added safety risks and may even mitigate side effects [12].

Smart medicine is also closing dangerous gaps in the current healthcare system. Today, misprescribing, poor adherence, and medical errors cost billions and harm millions. Smart medicines reduce these risks by delivering interventions in real time, guiding patients through biometric monitoring, optimizing dosing, and alerting providers of adverse events. Integration into clinical workflows and electronic health records ensures that digital data supports real-world decisions.

Moreover, smart medicines personalize treatment in ways traditional modalities cannot. They can dynamically adapt based on biometric feedback, behavioral engagement, or environmental triggers, offering a more continuous and precise therapy experience. By embedding clinical algorithms into interactive modules, these tools enable patients to actively participate in their care journey rather than passively receive instructions.

To truly unlock the transformative potential of smart medicines, we must envision a future of closed-loop care. Imagine these intelligent interventions deployed anywhere in America, continuously assessing a patient's health status and autonomously modulating both the intervention itself and any accompanying drug-based therapy. This closed-loop functionality has the potential to significantly reduce our reliance on costly and complex healthcare systems, numerous providers, and the sheer volume of traditional pharmaceuticals. In essence, smart medicine operating in a closed-loop fashion represents the missing link that can finally realize the promise of value-based care, an approach long discussed but difficult to implement at scale.

To support widespread clinical adoption, reimbursement models must evolve beyond fee-for-service. Smart medicines, especially when deployed as part of a closed-loop system, are capable not only of delivering interventions but also of continuously assessing patient status, adapting therapy, and optimizing outcomes in real time. This dynamic, feedback-driven model minimizes reliance on costly, fragmented provider infrastructure and reduces the need for redundant visits, diagnostics, or polypharmacy. When paired with traditional drug regimens, smart medicines can titrate both digital and pharmacologic interventions based on patient need, automatically, securely, and remotely. This is the missing link that value-based care has waited for. Instead of episodic, delayed responses to disease progression, smart medicines enable proactive, precision-guided health management from anywhere in America. These interventions can be reimbursed through outcomes-based contracts, formulary inclusion in Medicaid, Medicare, and commercial plans, and public-private partnerships that align incentives with clinical benefit. By decoupling care delivery from traditional site-based models, smart medicine transforms value-based care from a theoretical ideal into an operational reality.

While the potential is vast, implementation is not without challenges. Smart medicine initiatives must be supported by robust data privacy protections, digital literacy campaigns, and infrastructure investments to ensure nationwide reach. Some populations may lack access to smartphones or high-speed internet, and many clinicians may be unfamiliar with integrating digital therapeutics into their workflow. Addressing these disparities requires targeted training, device subsidies, expanded broadband, and seamless integration with existing EHR systems. Federal support will be essential to navigate these barriers and ensure no community is left behind.

The result is not just better care, it is financially transformative. Based on conservative modeling from the Centers for Medicare & Medicaid Services’ (CMS) National Health Expenditure data [13], we estimate that full-scale smart medicine deployment could generate more than $1 trillion in annual cost offsets across five high-burden domains by 2026 (Table 1). These domains were selected based on their contribution to preventable spending and well-documented responsiveness to behavioral or digitally enabled interventions. Below, we describe each category’s estimated savings and the underlying rationale.

**Table 1:** Estimated 2026 cost offsets from full-scale adoption of smart medicines in the United States. Conservative projections based on published healthcare spending benchmarks and modeled reductions from smart medicines across high-burden categories. These projections are directional estimates derived by the author using publicly available CMS data and conservative assumptions about intervention impact. They are intended to illustrate potential system-wide savings rather than serve as formal cost-effectiveness analyses. B: Billion; T: Trillion; SUD: Substance Use Disorder. Source: Reference [13]. Note: Created by the author.

Spend category	2026 estimated U.S. spend	Smart medicine impact	Projected savings
Chronic disease management	$4.2T	15-20%	$630B-$840B
Mental health and SUD	$360B	20-25%	$72B-$90B
Hospital readmissions	$65B	20%	$13B
Medication nonadherence	$650B	25%	$162.5B
Administrative inefficiency	$400B	10%	$40B
Total	-	-	$917.5B-$1.145T

Chronic Disease Management

Chronic conditions such as diabetes, heart disease, and obesity account for over 80% of total healthcare expenditures [13]. Smart medicines that improve day-to-day disease management, for example, through better diet, physical activity, medication adherence, behavioral reinforcement, and even modulation of brain circuits responsible for overeating, can reduce preventable comorbidities, complications, and emergency visits. We estimate savings at $630-840 billion (15-20%) based on the widespread impact of behavior-linked chronic illness and the ability of smart medicine to augment self-management across conditions.

Mental Health and Substance Use Disorders (SUD)

Smart medicines for depression, anxiety, post-traumatic stress disorder, and even opioid use disorder have demonstrated efficacy in randomized trials and are already FDA-authorized for deployment [1]. In the context of significant mental health provider shortages, smart medicines can serve as frontline or adjunct treatments, dramatically expanding access and reducing crisis-related utilization. We estimate savings at $72-90 billion (20-25%) based on their capacity to close care gaps, reduce hospitalizations, and improve recovery outcomes.

Hospital Readmissions

Many readmissions are preventable when post-discharge adherence is optimized. Smart medicines can support real-time symptom tracking, prompt therapeutic engagement, and early intervention, avoiding the common “failure-to-fill” and “failure-to-follow-up” gaps in the current system. By enabling digital follow-up, reinforcement of discharge instructions, and automated coaching, smart medicines are projected to save $13 billion (20%) annually.

Medication Nonadherence and Misuse

Nonadherence and misuse contribute to nearly $650 billion in wasted spending annually [13]. Smart medicines support adherence by providing real-time guidance, tailored behavioral support, reminders, biometric integration, and personalized feedback once a therapeutic alliance is established. These tools enhance both the appropriateness and continuity of pharmacologic regimens, with estimated annual savings of $162.5 billion (25%).

Administrative Inefficiency

Administrative waste is driven by manual processes, fragmented systems, and redundant tasks. Smart medicines reduce these burdens by digitizing components of care delivery, follow-up, remote monitoring, and documentation. By streamlining communication and automation, they can reduce friction in the system and save an estimated $40 billion (10%) per year.

These domains offer a realistic, evidence-based path to $1 trillion in cost containment and value creation, not including additional, substantial gains from increased workforce productivity, reductions in caregiver burden, and avoidance of long-term disability and care. For example, untreated or poorly managed chronic and mental health conditions are leading causes of absenteeism and presenteeism, costing employers hundreds of billions annually. Similarly, the stress and unpaid labor borne by family caregivers, often due to a lack of accessible behavioral health or chronic disease support, impose additional hidden costs. Smart medicines, by offering scalable tools for early intervention, continuity of care, and behavioral stabilization, not only reduce formal healthcare costs but also relieve societal and economic pressures that were not factored into the current calculation.

More importantly, they address systemic goals. Smart medicines advance nearly every HHS priority, from improving maternal health and behavioral health to managing multimorbidity, reducing healthcare disparities, and promoting access in rural and underserved populations (Table 2) [2]. They also directly align with the vision set forth by the U.S. President’s Make America Healthier Again (MAHA) Commission, established by executive order [14], which calls for bold, system-wide action to reverse the chronic disease crisis and end childhood illness. Smart medicines deliver on this mandate through closed-loop therapeutic systems that not only deliver care but also continuously monitor, adapt, and optimize it, reducing reliance on fragmented infrastructure, expensive drugs, and delayed interventions. These strategies embody MAHA’s priorities by restoring scientific integrity, enabling lifestyle-based care, and transforming health outcomes through evidence-based innovation that reaches all Americans. By embedding closed-loop therapeutic systems directly into the lives of Americans, we can advance MAHA’s core goals: reversing chronic disease, safeguarding children’s health, and building a healthcare system that delivers outcomes.

**Table 2:** Strategic alignment of smart medicines with 2025 Health and Human Services (HHS) and Make America Healthier Again (MAHA) priorities. Mapping of how prescription digital therapeutics and drug-software combinations, smart medicines, support U.S. Health and Human Services (HHS)’s core pillars under the Make America Healthier Again (MAHA) initiative. PTSD: Post-Traumatic Stress Disorder. Source: References [2, 14]. Note: Created by the author.

HHS/MAHA Priority	Smart Medicine Contributions (Examples)
Chronic disease epidemic	FDA-cleared digital therapeutics for type 2 diabetes, obesity, hypertension, migraine, and chronic insomnia
Behavioral health	FDA-cleared digital therapeutics for major depressive disorder and PTSD, deployed amid severe psychiatric provider shortages
Health equity	Technology-enabled care for rural, disabled, underserved, and low-income populations
Maternal and family health	Digital support tools for postpartum depression, parenting challenges, and sleep health
Emergency preparedness	Digital interventions for PTSD and trauma recovery tailored to veterans, first responders, and crisis-affected populations
Lowering drug costs	Digital titration, adherence optimization, and behavioral support to reduce unnecessary prescriptions and polypharmacy
Food and nutrition security	App-based coaching to promote healthy eating, reduce cravings, and reinforce sustainable lifestyle behaviors

A win for America’s innovation

No country is better positioned than the United States to lead this transformation. Smart medicines are a homegrown innovation: conceived by American scientists, developed by American biotechs, cleared by American regulators, and delivered by American clinicians. This is where our world-class biomedical research ecosystem converges with cutting-edge software engineering, AI, and neurobehavioral science to deliver patient-level precision at scale.

Unlike traditional medications, smart medicines are powered by software, not biochemistry. They are unconstrained by raw materials or manufacturing bottlenecks and can be deployed instantly across the country, updated in real time as new evidence emerges. These interventions offer full auditability, dynamic personalization, and continuous therapeutic engagement. With each use, they generate data that drives real-world validation, accelerates innovation, and creates an evolving cycle of evidence-based care, what we call “living medicine.”

This innovation pipeline doesn’t just heal, it hires. Entire sectors of the American economy, from AI and cloud computing to behavioral science and cybersecurity, stand to benefit from the smart medicine revolution. Intellectual property stays domestic. High-value jobs are created. Economic value is retained and reinvested. And as global demand for digital health accelerates, smart medicines could become America’s next great export, just as semiconductors and the internet once did. In fact, while other nations scramble to retrofit outdated health systems, the U.S. has the opportunity to set the global gold standard for regulated, AI-enabled software therapeutics.

The U.S. can reassert its dominance as the world’s health technology superpower. American companies would lead ethically and clinically, setting the standards others follow. Our patients would benefit first. Our workforce would fuel the expansion. Our leadership in digital medicine would not just reflect American innovation, it would restore American health, prosperity, and global standing.

A win for America

Smart medicines deliver on all fronts. They are a rare intervention that improves care while lowering cost, scaling with evidence and political viability. Equity advocates see a tool to close gaps. Technologists see a platform for health transformation. Fiscal conservatives see over $1 trillion in projected savings. For all stakeholders, this is not just innovation, it’s progress with purpose.

We’ve mobilized this kind of transformation before. Operation Warp Speed proved that America can unite science, industry, and government to drive unprecedented innovation at scale [15]. The HITECH Act, which catalyzed near-universal adoption of electronic health records (EHR), showed how federal levers, financial incentives, regulatory standards, and clear timelines, can reshape the clinical landscape [16]. Today, the U.S. leads the world in EHR infrastructure. Meanwhile, Germany’s EHR system is stalled [17], France’s suffers from fragmentation [18], and Canada’s progress remains patchy [19]. These examples remind us: universal coverage alone does not enable digital transformation, national strategy does.

It’s time for that strategy. It’s time for Operation Smart Medicine. This bold initiative would coordinate federal agencies to fast-track the development, authorization, reimbursement, and equitable deployment of evidence-based digital therapeutics (Table 3). The White House could establish a Presidential Task Force on Smart Medicine, embedded within HHS and reporting directly to the Executive Office of the President. Congress could authorize multi-year appropriations for infrastructure, clinical validation, and access expansion. CMS could launch value-based pilots and national coverage pathways. FDA could finalize its Prescription Drug Use-Related Software (PDURS) framework and accelerate modular clearances for drug-software combinations [20]. The Office of the National Coordinator (ONC) could enforce interoperability, certification, and cybersecurity standards. Veterans Affairs and Indian Health Services could pioneer deployment in federal care systems.

**Table 3:** Federal agency and office roles in Operation Smart Medicine, modeled on Operation Warp Speed. This author-created table outlines how U.S. federal agencies and offices that played key roles in Operation Warp Speed can assume analogous responsibilities to support the national deployment of smart medicines. These roles span regulation, funding, logistics, security, and public health integration to accelerate the adoption of evidence-based digital therapeutics. Source: reference [15]. Note: Created by the author.

Federal Agency/Office	Role in Operation Warp Speed	Parallel Role in Operation Smart Medicine
Executive Office of the President (EOP)	Coordinated national policy and public communication via Coronavirus Task Force	Drive whole-of-government coordination through a Presidential Task Force
U.S. Department of Health and Human Services (HHS)	Led coordination, funding, regulatory oversight, and public health response	Coordinate national digital health policy, funding, and health equity integration
National Institutes of Health (NIH)	Led vaccine research and academic partnerships (e.g., Moderna)	Fund clinical trials, digital engagement research, and implementation science
Biomedical Advanced Research and Development Authority (BARDA)	Funded and procured vaccines and therapeutics	Support rapid development and deployment of high-impact smart medicines
U.S. Food and Drug Administration (FDA)	Provided emergency use authorizations and safety oversight	Accelerate regulatory review of smart medicines, especially drug–digital combinations
Centers for Disease Control and Prevention (CDC)	Managed vaccine distribution and public health surveillance	Integrate smart medicines into public health guidelines and prevention programs
U.S. Department of Defense (DoD)	Oversaw logistics, supply chain, and deployment operations	Manage logistics for digital infrastructure, provider readiness, and deployment
Office of the Assistant Secretary for Preparedness and Response (ASPR)	Led emergency preparedness and BARDA coordination	Align preparedness strategies with chronic disease and digital health capabilities
General Services Administration / U.S. Department of the Treasury (GSA/Treasury)	Streamlined contracts, procurement, and funding flows	Facilitate scalable procurement and tax credits for health technology adoption
Federal Emergency Management Agency (FEMA)	Supported distribution logistics and emergency site setup	Deploy digital tools in disaster response and emergency chronic care management
U.S. Public Health Service Commissioned Corps (USPHS)	Provided staffing for national vaccine administration	Provide clinical capacity to deliver and educate on smart medicine access
U.S. Department of Energy (DOE)	Contributed supercomputing for modeling and vaccine optimization	Support modeling, infrastructure optimization, and secure digital platforms
U.S. Department of Homeland Security (DHS)	Secured national infrastructure and supported cybersecurity	Protect national digital health systems and ensure emergency resilience
U.S. Department of Education (ED)	Not directly involved but relevant to public awareness and access	Promote digital literacy and integration in school-based health initiatives
National Science Foundation (NSF)	Provided foundational science support for research and analytics	Fund foundational research in behavioral science, AI, and digital efficacy
U.S. Department of Justice / Office for Civil Rights (DOJ/OCR)	Issued HIPAA flexibilities and data privacy guidance	Ensure compliance with civil rights and patient privacy in digital implementation
Federal Communications Commission (FCC)	Enabled broadband and emergency telehealth access	Expand broadband and telehealth capacity to reach underserved populations

To support scale, trust, and system-wide adoption, a Smart Medicine National Accelerator could serve as the backbone of this initiative, curating evidence, standardizing implementation, and fostering public-private collaboration. Modeled on successful efforts like National Institutes of Health's (NIH) All of Us [21], this framework would balance innovation with equity through transparent, stakeholder-informed governance. A national formulary of FDA-authorized smart medicines would provide payers and providers with a trusted, up-to-date reference for coverage, reimbursement, and clinical integration. Beyond cataloging, the Accelerator would support integration by standardizing evidence reporting, promoting shared infrastructure for deployment, and enabling the aggregation of independently developed products into a coherent, interoperable ecosystem. Patients, especially those served by the VA, Indian Health Service, Medicaid, and Medicare, could gain access at little or no out-of-pocket cost. Robust governance structures will be essential to address intellectual property rights, data ownership, and the stewardship of shared clinical algorithms, ensuring that innovation incentives are preserved while enabling equitable, secure, and accountable system growth.

Operation Smart Medicine is more than a health initiative, it may be a political breakthrough. It fulfills core goals of the MAHA Commission by reversing chronic disease, protecting children’s health, and realigning the healthcare system toward outcomes [14]. Democrats will champion expanded access and digital empowerment. Republicans will see deregulation, innovation, and budgetary discipline. Independents will see functional government that delivers for real people. In a fractured political era, few policies unite public health, fiscal sustainability, and national competitiveness this effectively. Smart medicine offers a credible, scalable pathway to align clinical innovation with national health reform, if supported by the right regulatory, reimbursement, and distribution infrastructure. Then, smart medicine would not just be smart policy, but the future of medicine.

Conclusion

I have spent my career caring for Americans across every geography and socioeconomic divide. I have trained the physicians who will inherit this system and helped develop the therapies that can transform it. What we need now is alignment between science and policy, innovation and implementation, care and common sense.

Smart medicine is not a distant future, it is a proven present waiting to be scaled. It is the clearest path to reversing chronic disease, empowering patients, and restoring the health of a nation that has too long accepted dysfunction as destiny. We have the technology. We have the precedent. We have a federal mandate in MAHA and a moment that demands action.

Let us lead, not with inertia, but with innovation. Let us act, not incrementally, but boldly. Let us make America healthy again by going digital, going decisive, and putting people before process, outcomes before bureaucracy, and progress before partisanship.
